# Can the Wolf (*Canis lupus*) Thrive in Highly Anthropised Lowlands? First Habitat Suitability Analysis of the Po Plain, Italy

**DOI:** 10.3390/ani15040546

**Published:** 2025-02-13

**Authors:** Luca Fardone, Martina Forlani, Luca Canova, Matteo De Luca, Alberto Meriggi

**Affiliations:** 1 Department of Earth and Environmental Sciences, University of Pavia, 27100 Pavia, Italy; martinaforlani.biologa@gmail.com (M.F.);; 2 Department of Chemistry, University of Pavia, 27100 Pavia, Italy; 3 For-Nature S.r.l., Via Ciconi 26, 33100 Udine, Italy

**Keywords:** *Canis lupus*, MaxEnt, distribution, human impact, behavioural plasticity

## Abstract

Coexisting with large carnivores in human-dominated European landscapes is a highly relevant and current challenge. Over the last two centuries, the wolf (*Canis lupus*) population in Europe has experienced a significant decline, primarily due to direct human persecution. However, recent conservation policies, combined with the species’ remarkable ecological flexibility, have enabled a rapid recovery. This process, which now also extends to densely populated areas, is exemplified by the ongoing expansion across the Po Plain, one of Europe’s most heavily human-dominated landscapes. Our study aims to provide the first assessment of habitat suitability for wolf presence in the Po Plain. Using a ten-year dataset of wolf occurrences (2015–2024), we evaluated the influence of several environmental and human-related factors by applying habitat suitability models with the Maximum Entropy algorithm (MaxEnt). The goal was to quantify potentially suitable habitats within the plain and to identify the factors that could either facilitate or constrain wolf presence. The results showed that approximately half of the Po Plain is suitable for wolf presence. Among anthropogenic variables, “urban areas” was the only factor that significantly and negatively affected habitat suitability, while other variables had negligible impacts. This underscores the species’ remarkable ecological and behavioural adaptability. By investigating wolf distribution in one of Europe’s most anthropised regions, this study aims to shed light on the species’ ability to thrive in human-altered landscapes, contributing to ongoing conservation efforts and informing future strategies for coexistence in highly populated lowland ecosystems.

## Simple Summary

Over the last two centuries, wolf populations in Europe nearly disappeared due to human actions, but thanks to conservation efforts and the species’ ability to adapt, they are making a remarkable comeback. This recovery is now extending into densely populated areas, such as the Po Plain in northern Italy, one of Europe’s most heavily modified landscapes. The goal of this study was to quantify potentially suitable habitats within the plain and identify the factors that could facilitate or constrain wolf presence. We found that about half of the Po Plain offers suitable habitats for wolves. Interestingly, urban areas were the only significant human factor negatively impacting wolf presence, while other factors had little influence, highlighting the species’ exceptional adaptability. By examining wolf distribution in such a challenging environment, our research underscores the potential for coexistence between humans and wildlife in even the most altered landscapes. These findings provide valuable insights for improving conservation strategies and promoting coexistence between people and predators in densely populated areas.

## 1. Introduction

In recent decades, the wolf (*Canis lupus*) has made a significant comeback across Europe [1]. At the beginning of the 20th century, wolf populations were nearly eradicated from most of Western Europe, including the Alps [2], surviving only in isolated regions [3,4]. In Italy, the species suffered a severe decline, reaching a historic minimum in the 1970s, when fewer than 100 individuals remained in fragmented areas in the Central and Southern Apennines [4,5]. This decline was driven by several factors, including direct persecution, habitat destruction, and a reduction in prey species caused by expanding agriculture and deforestation [2,6]. However, the implementation of strict legal protection, along with changes in land use practices that led to habitat regeneration—such as depopulation of rural areas [7]—have allowed this species to reclaim much of its historical range [8,9]. Since the early 1980s, the population has steadily recovered and rapidly recolonised the northern Apennines [8]. As the population expanded, wolves gradually moved into Western Alps, marking the re-establishment of the species in this region after nearly a century of absence [8]. Simultaneously, recolonization of the Eastern Alps has been supported by dispersing individuals from the Dinaric-Balkan population, facilitating a broader recolonization of the entire Alpine region [10]. Currently, the species is protected in Europe under the Bern Convention and the Habitats Directive (92/43/EEC). However, a revision of the legal status of the wolf is currently underway, aiming to downgrade its classification from “strictly protected species” to “protected species”. This change could have practical implications, particularly regarding the possibility of applying derogations from the Directive for wolf management in different EU member states.

In Italy, according to the latest national monitoring, the wolf population has been estimated to be approximately 3300 individuals (range: 2945–3608) [11]. Once confined to forested and mountainous regions, wolves are now expanding into new and unexpected territories, including areas characterised by high human density and intensive land use [1]. A striking example of this phenomenon is the recolonization of the Po Plain in Northern Italy [9], one of Europe’s most densely populated and agriculturally intensive regions. This area does not appear to represent a suitable habitat for large carnivores due to its high population density, minimal forest cover, and intensive agricultural practices. The landscape is heavily fragmented, with large tracts of agricultural land interspersed with urban areas and infrastructure, creating significant barriers to wildlife movement. Thus, habitat fragmentation can limit the establishment of stable wolf territories and pack formation. Additionally, traffic collisions represent an important cause of mortality for wolves [12,13]. However, recent studies across Europe have demonstrated that wolves are also expanding their range in urbanised areas by utilising human-made structures, such as roads and agricultural fields, as movement corridors [14,15,16,17]. They appear to adjust their behaviour to avoid human activity, often adopting a more nocturnal pattern [18]. This behavioural flexibility allows them to inhabit a broader array of habitats than previously assumed, enabling survival in areas with high human disturbance. Nevertheless, studies conducted in these specific contexts remain limited. Indeed, much of the research aimed at identifying the factors driving wolf distribution and habitat suitability typically focuses on areas with low human densities, high forest cover, or mountainous land that provides adequate refuge [15,19,20,21,22,23]. In the Northern Apennines, however, where wolves seem to have already occupied all available lands [24], recent expansion has also reached the more urbanised areas of the Po Plain, where environmental conditions differ substantially. This setting exemplifies the species’ remarkable ecological and behavioural plasticity, allowing it to adapt to markedly different environments.

This study aims to provide the first detailed assessment of habitat suitability for wolves in the Po Plain, focusing on both environmental and human-related factors that influence their presence, and quantifying the extent of suitable habitat in this region. By analysing a ten-year dataset (2015–2024) of wolf occurrences, collected through various methods including camera trapping, scat sampling, telemetry, and citizen reports, we formulated species distribution models using the Maximum Entropy algorithm (MaxEnt), often used for elusive species like wolves due to its ability to model habitat suitability without the need for absence data [23,25,26,27].

Ultimately, our goal is to assess whether the highly modified landscape of the Po Plain is suitable for wolf presence and identify the critical factors influencing habitat suitability for the species. In light of growing wolf populations across Europe, this region serves as a unique case study of how large carnivores adapt to human-dominated environments. Understanding the dynamics of their recolonization in such regions will not only inform local conservation and management efforts but also contribute to broader discussions on human–wildlife coexistence in the modern era.

## 2. Materials and Methods

### 2.1. Study Area

**Figure 1:**
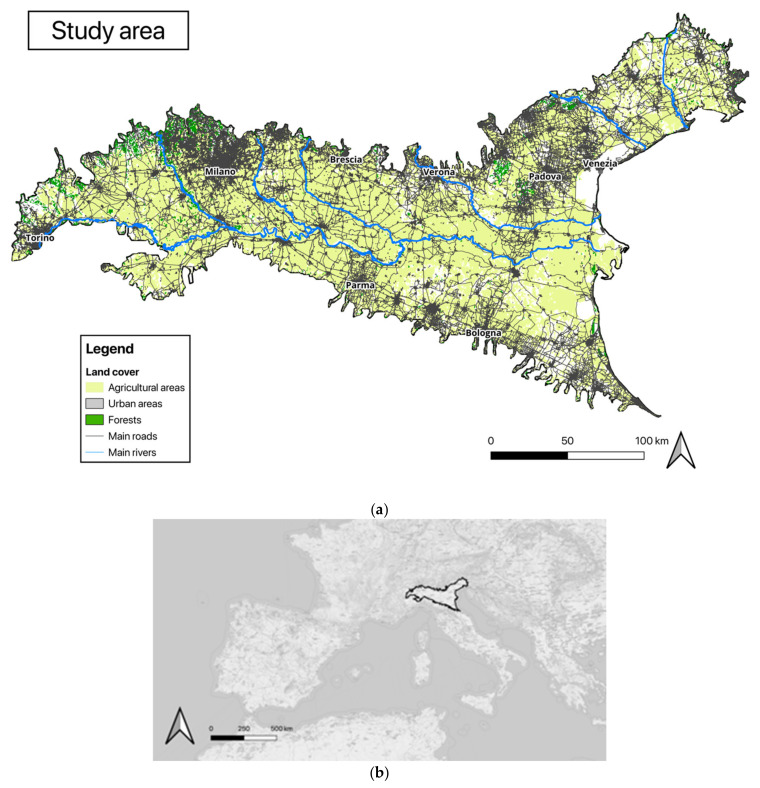
(**a**) Study area in the Po Plain showing land cover classes along with main rivers and roads; (**b**) study area location in Europe.

The study area encompassed the Po Plain, a vast region extending over approximately 45,000 km^2^ in northern Italy (Figure 1). This area includes several administrative regions: Piedmont, Lombardy, Emilia-Romagna, Veneto, and Friuli-Venezia Giulia. Characterised by intensive agricultural, industrial, and logistical activities, the Po Plain is one of the most densely populated regions in Europe, with a total population exceeding 20 million residents and an average population density of 450 inhabitants per km^2^, more than double the national density (ISTAT census of 2021). The landscape is predominantly open and flat (max elevation: 300 m a.s.l.), with 81% of the plain exhibiting low to medium-low levels of landscape diversity [28]. Most residual wood patches (mean size: 4.5 ha) are located along the main tributaries of the Po River [29]. In the entire Po Plain, only a small number of natural woodland fragments remain and, among these, only eleven exceed 200 ha in size [30].

Urban areas consist of large cities, including Milan, Turin, and Bologna, as well as thousands of small towns and villages interspersed with agricultural lands and a few small wooded patches. Human activities, such as urban expansion, agricultural practices, and infrastructure development, have significantly altered the natural landscape over the centuries (Table 1).

Despite its high level of human modification, habitats such as riparian forests and agroforestry areas provide suitable habitat and potential refuges for wildlife. The area hosts a diverse mammalian fauna, well-suited to the varied landscapes ranging from rural to anthropised environments. Among these, the roe deer (*Capreolus capreolus*), wild boar (*Sus scrofa*), and the fallow deer (*Dama dama*) thrive in the countryside and wooded areas scattered throughout the plain. However, it should be noted that the distribution of these ungulates is not uniform across the entire Po Plain but is rather restricted to specific areas. Moreover, species such as the European hare (*Lepus europaeus*) and the eastern cottontail rabbit (*Sylvilagus floridanus*) are found in several habitats, ranging from woodlands to open fields. Additionally, it is important to highlight the presence of the coypu (*Myocastor coypus*) in water bodies and irrigation channels across the area. The coypu is an invasive alien species with an expanding distribution in the region, posing ecological challenges due to its impacts on native flora and human activities. The presence of these mammals potentially renders this territory suitable for wolves from a trophic perspective, but anthropogenic pressure and habitat fragmentation remain extremely high. Additionally, it is important to note the widespread presence of intensive livestock farming, particularly involving pigs and cattle. However, these livestock are confined within facilities, and the husbandry practices employed in the area prevent their accessibility to wolves, rendering them an irrelevant resource for wolves.

### 2.2. Wolf Presence Data

We collected georeferenced wolf occurrence data within the study area during a ten-year period (2015–2024); they were mostly collected as occasional presence data because of the absence of specific monitoring projects. The samples included in our dataset were all validated by a recognised expert in wolf ecology and scat analysis, A.M., who has extensive experience in the field. This rigorous validation process ensured data reliability and minimised the risk of misidentification, ensuring high-quality data despite the diverse sources from which they originated. It is important to note that during the sampling phase, it was not possible to distinguish between resident wolves and dispersers. Therefore, the collected data include presence points for both resident individuals and solitary dispersing individuals. The total dataset amounted to 651 presence records. These were scat samples (n = 208), camera trap pictures (n = 168), telemetry data from a radio-collared wolf (n = 57), and direct observations accompanied by video/photographic evidence (n = 136). Additionally, reports of wolf carcasses from road collisions (n = 33) and newspaper articles identifying the exact location through videos or images (n = 49) were also included. Camera traps were opportunistically deployed in 111 different locations within the study area between January 2018 and December 2023.

We used a 5 × 5 km grid to record the Universal Transverse Mercator (UTM) coordinates of all wolf signs, consistent with other habitat suitability studies conducted for the species in Northern Italy [19,31]. To reduce inaccuracies caused by spatial autocorrelation due to non-uniform sampling efforts, duplicates within cells were removed, and a single occurrence was randomly retained from each cell containing more than one occurrence [32], resulting in 155 presence records.

### 2.3. Environmental and Human Related Variables

We used five categories of environmental covariates as potential predictors of wolf habitat suitability: (1) habitat composition, divided into seven land-use categories based on wolf ecology: wood cover, open cultivated areas, closed cultivated areas (e.g., agroforestry areas), open natural areas, shrublands, urban areas, and other (Table 1. Supplementary Material—Table S1); (2) distance from rivers; (3) distance from protected areas; (4) distance from main roads, and (5) human disturbance (human population density and artificial light at night). The selection of variables was based on their ecological relevance to the species, as indicated in the literature, and was adapted to our specific environmental context. The land cover categories were obtained through land cover data [33], with a resolution of 100 m, and their proportion (%) within each grid cell was calculated. Roads were derived from OpenStreetMap (OSM) data [34], while rivers and protected areas were obtained from the INSPIRE Geoportal [35]. Human population density and artificial light at night were extracted from Global Terrestrial Human Footprint Maps for 2009 [36] with a resolution of 1km. All variables were processed using GIS software (QGIS version 3.34.2-Prizren) [37] and rescaled to a resolution of 5 × 5 km using the WGS 84 UTM 32N projection (EPSG: 32632).

We used Pearson’s correlation coefficient to evaluate the correlation between the variables and reduce multicollinearity, which can lead to overfitting and decrease model accuracy [38]. When two environmental variables had a correlation coefficient (r) ≥ |0.6|, we selected only one for inclusion in the model, based on its importance in the literature on wolves (Table 2). Specifically, the analysis showed a significant correlation between artificial light at night and population density; therefore, we included only population density in the final analyses. All data analyses were performed using R Studio (RStudio 2022.07.2) [39].

### 2.4. Species Distribution Model

We modelled habitat suitability for wolves using MaxEnt (Version 3.4.4) [40], a machine-learning program based on maximum entropy algorithm. MaxEnt is widely acknowledged for its robustness and accuracy in characterising species–habitat associations from their presence background data [41]. This method estimates habitat suitability for a species based on environmental constraints by seeking a marginal suitability function for each variable [42]. One advantage of MaxEnt is its independence from absence data, which are often scarce for elusive species like wolves and may include suitable yet uncolonised areas [42]. Moreover, MaxEnt has been shown to outperform other presence-only modelling techniques [43] and remains effective with small sample sizes [44]. Its reliability has been affirmed in various contexts, such as predicting the outcome of invasive species introductions and identifying new presence localities for poorly known species [45,46]. The model incorporates two categories of data inputs: locations where the presence of a species has been recorded (presence-only data) and environmental layers for the study area. To create the habitat suitability map, MaxEnt evaluates the environmental conditions of the presence data against a background set of random data points, which reflect the environmental conditions available throughout the study region. MaxEnt’s utilisation of the maximum entropy principle enables us to evaluate grid cell suitability based on environmental variables, providing a suitability map ranging from 0 to 1 [42].

We performed 100 bootstrap replications, using 75% of the presence points for training and the remaining 25% for testing. A set of 10,000 background locations was randomly generated across the study area. For all models run in this study, we used the default settings for the regularisation parameter. The feature classes included linear, quadratic, product, threshold, and hinge. Other settings were maximum iterations of 500, a convergence threshold of 0.00001, an adjusted sample radius of 0, a default prevalence of 0.5, and the output “logistic”. The threshold rule was set to the 10th percentile training presence, frequently used in studies relying on datasets collected over a long time by various observers and techniques [47,48]. This threshold was used to reclassify the model into a binary map. Models were evaluated based on ecological realism [49] and using the area under the Receiver Operating Characteristic (ROC) curve (AUC) [50] as well as the True Skill Statistic (TSS) [51]. AUC values range from 0.5 (random distribution) to 1 (perfect discrimination). Models with AUC > 0.75 were considered to have high discrimination performance [50].

## 3. Results

**Figure 2:**
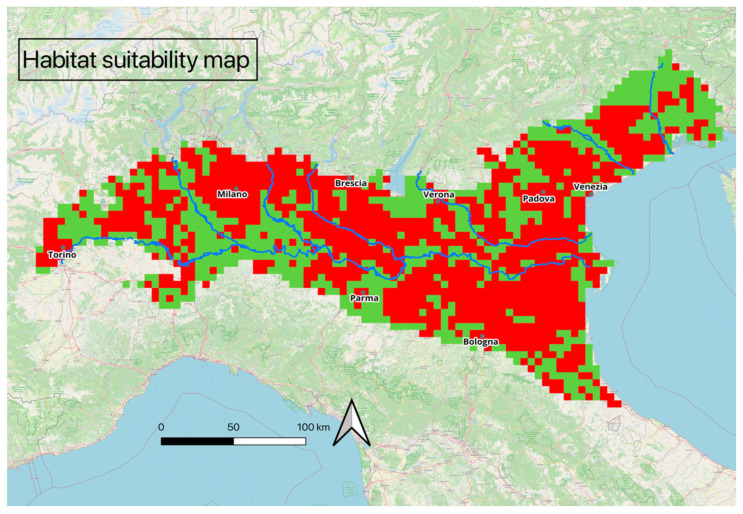
Binary habitat suitability map for wolves in the Po Plain. Suitable cells are shown in green, and unsuitable cells are shown in red.

The model predicted a total of 715 cells with suitable habitat for wolves, corresponding to an area of 17,875 km^2^, which represents 40.51% of the Po Plain’s surface area (Figure 2). The calculated average (±SE) AUC for the replicate runs was 0.893 (±0.016) and the TSS was 0.49, indicating that the model had reasonable predictive power.

Based on permutation importance, the most important variable in this model was the distance to woods (14.4%), followed by the proportion of urban areas (14.2%), proportion of open cultivated areas (11.9%), proportion of closed cultivated areas (10.5%), distance to rivers (8%), and wood cover (6.2%). Population density and distance to main roads all had importance values of less than 6% (Table 3).

**Figure 3:**
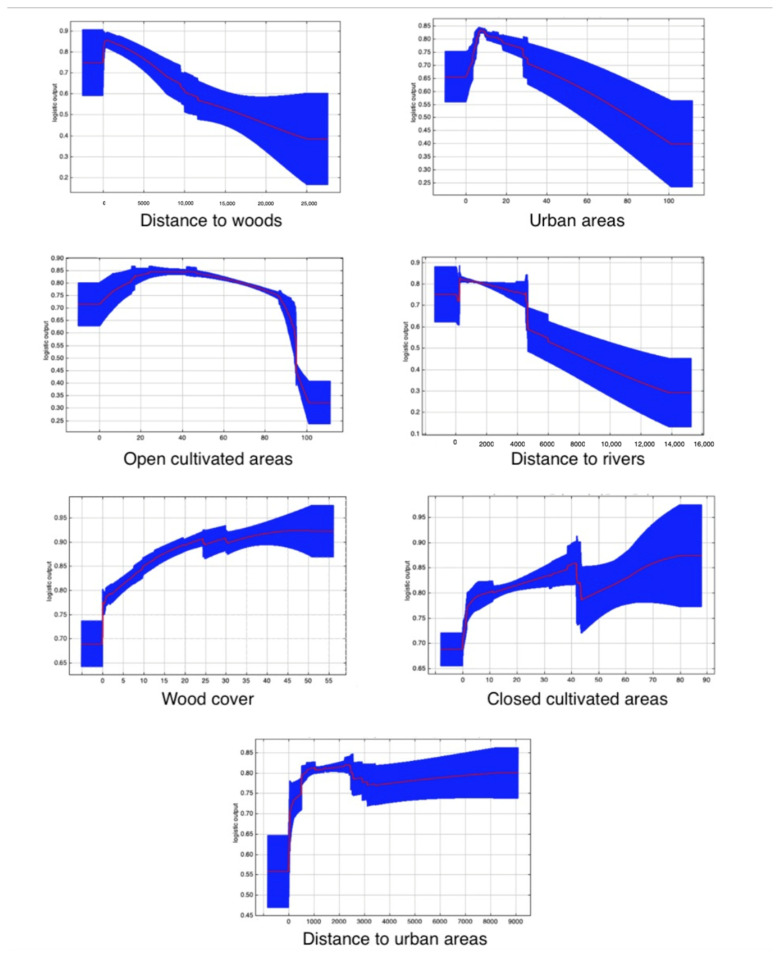
Response curves for most important environmental variables in the model. The curves show how the logistic prediction changes with each environmental covariate. Red lines represent the average trend for the variable considered, while the blue shading represents the standard deviation from 100 bootstrapped replications.

The variables “wood cover”, “closed cultivated areas”, and “distance to urban areas” had positive effects, while “distance to woods”, “distance to rivers”, “urban areas”, and “open cultivated areas” had negative effects on wolf habitat suitability (Figure 3). Notably, when the proportion of “open cultivated areas” exceeded 40%, suitability decreased and, beyond 80%, this decrease became substantial. Additionally, suitability reached its maximum value at a distance of just 1km from urban areas.

## 4. Discussion

In this study, we present the first assessment of habitat suitability for wolves in the Po Plain. According to the MaxEnt model, approximately 40% of the Po Plain has been identified as suitable for the species. This finding is particularly significant given the environmental characteristics of the study area, especially in comparison to other European contexts where wolves are typically studied. In fact, the Po Plain is a unique environmental setting, being open, flat, and representing one of the most densely populated, industrialised, and anthropogenically altered regions in Europe. The presence and reproduction of wolves in this area highlight their remarkable behavioural adaptability, which enables them to thrive even in highly modified landscapes [24,52]. The colonisation of such highly anthropised lowland areas is the result of a gradual process in which the species progressively occupied all available free areas in mountainous and hilly environments [24]. During the early stages of this process, when large portions of natural or semi-natural land were still unoccupied, human presence likely played a crucial role in the selection of territories. As suggested by Zanni et al. [14], there was a general tendency to avoid highly anthropised areas in favour of territories with forest cover and reduced human disturbance, leading many researchers to assess exclusively forested areas with low human impact as suitable for wolves [19,21,27,53,54,55,56,57]. However, this tendency to avoid humans only delayed the recolonization of human-dominated landscapes [14]. Indeed, once areas with low human impact were occupied, dispersing wolves in search of new, unclaimed territories had to adapt to highly modified habitats with a strong human presence, such as the Po Plain [24]. Wolves are likely to thrive wherever food is available and human persecution is minimal, even in human-dominated landscapes [15,58,59,60].

Recent studies examining ongoing recolonization in human-dominated landscapes suggest that anthropogenic disturbance is less of a limiting factor for wolf presence [14,24,61]. Within this context, our results showed that the only anthropogenic variable significantly and negatively affecting habitat suitability was “urban areas”, while other factors had negligible impacts. Our findings indicate that human-related factors such as population density and road density do not negatively impact habitat suitability. The only clear limiting factor appears to be direct human presence, characteristic of urban areas, which are identified as highly unsuitable, but even at short distances from urban settlements (around 1 km), the land becomes suitable for the species. This result contrasts with several studies that identified anthropogenic features such as roads as major disturbances for wolves [21,62]. However, those studies focused on natural or semi-natural areas, typically characterised by much lower human density and disturbance compared to the Po Plain. In a landscape where infrastructure like roads and railways are pervasive, the exclusion of such elements from a wolf’s territory, as happens in other contexts, would mean disregarding large portions of available land. Accordingly, our findings are not entirely unexpected but rather highlight the species’ remarkable ecological flexibility. Recent research has shown that wolves in human-dominated landscapes exhibit significant plasticity to minimize risks, secure food sources, and maintain breeding success [27]. In areas where ungulates are scarce, they adapt by hunting smaller prey or scavenging [63,64]. Other studies have shown that wolves adjust their behaviour based on the spatial and temporal patterns of human presence, responding in ways that align with the expected seasonal and daily fluctuations in human activity [65]. Moreover, territory sizes also vary based on resource distribution, with smaller packs forming in fragmented habitats and larger packs in resource-rich areas [66]. All these factors highlight the ecological and behavioural plasticity of wolves, enabling them to thrive even in highly altered environments.

As expected, among the other selected variables, the presence of wooded areas, though limited in extent, plays a crucial role, as low wood cover or significant distances from wooded areas lead to a considerable reduction in habitat suitability. This result is consistent with the literature, which underscores the importance of wood cover for resting and reproductive activities [60,67], particularly in densely populated regions, where woods and shrublands are less exposed to direct human presence. In particular, the forest corridor along the Ticino River, a significant portion of that along the Po River, and much of the Friuli Plain appear to be the most suitable areas for wolves in Po Plain. We also found that agricultural areas are generally suitable for wolf presence, except when there is an extremely high proportion of open agricultural land, highlighting the predator’s preference for at least partially covered environments [58]. Similarly, the positive effect of closed cultivated areas and agroforestry areas can be explained, considering also that these landscapes typically offer higher prey densities compared to fully open cultivated areas.

Our analysis, however, lacks one key factor influencing habitat suitability: prey availability. We did not have access to a dataset describing density or presence/absence of prey species. In the study area, the distribution of wild ungulates is fragmented and limited to certain zones. However, the high density of coypus (*Myocastor coypus*), which are widely distributed throughout the Po Plain [68,69], seems to play a crucial role in the rapid recolonization by wolves. Recent studies in the Italian lowlands reveal that coypus and other rodents may regularly be consumed by wolves in specific environmental contexts [63,64], especially when wild ungulates are less available. Further research into the wolf diet in this area would provide valuable insights into their ecological adaptations.

Another important factor to consider, which could affect species distribution models, is the dispersal phase. During this phase, some individuals may cross unsuitable areas, potentially introducing bias if sampled. This could result in identifying areas as suitable, even if they cannot support critical life stages, such as reproduction, which requires specific environmental conditions that appear to be rare in the Po Plain. Developing a suitability model based on breeding sites would be valuable to eliminate any potential bias of this nature and to better identify areas truly capable of sustaining a stable wolf population.

## 5. Conclusions

This study provides a valuable first assessment of habitat suitability for wolves in the Po Plain, one of the most densely populated and anthropogenically altered regions in Europe, identifying significant portions of the region as suitable for the species. By modelling habitat suitability for wolves in such a unique and challenging environment, this research contributes to the growing body of literature on wolf conservation in human-dominated landscapes.

Our results highlight the remarkable ecological flexibility of wolves, which can colonise and reproduce in highly anthropogenic environments, where human presence and infrastructure are widespread. These findings inform local conservation and management strategies while also providing valuable insights into how wolves can adapt even to the most heavily human-exploited environments. Understanding the wolves’ ability to navigate these anthropised landscapes is critical for ensuring their continued survival in an era of rapid environmental change.

## Figures and Tables

**Table 1:** Land cover classes and corresponding area (expressed in km^2^ and %) within the Po Plain.

Habitat Type	Area km^2^ (%)
Open cultivated areas	29.610 km^2^ (66.0%)
Urban areas	5.862 km^2^ (13.1%)
Closed cultivated areas	5.643 km^2^ (12.6 %)
Woods	1.404 km^2^ (3.1%)
Shrublands	374 km^2^ (0.8%)
Open natural areas	353 km^2^ (0.8%)
Other	1.609 km^2^ (3.6%)

**Table 2:** Variables used to model habitat suitability in the Po Plain, with expressed measure unit, description, and reference.

Covariate	Unit of Measure	Description
Human population density	log-transformed	3.333 × log (population density + 1) with continuous values (0–10)
Wood cover	%	Proportion of forest cover in the grid cell
Open cultivated areas	%	Proportion of open/semi-open cultivated areas in the grid cell
Closed cultivated areas	%	Proportion of closed cultivated areas (i.e., land principally occupied by agriculture with significant areas of natural vegetation or agroforestry areas) in the grid cell
Open natural areas	%	Proportion of open natural areas in the grid cell
Shrublands	%	Proportion of shrubs in the grid cell
Urbanised areas	%	Proportion of urban areas in the grid cell
Distance to the closest road	m	Distance between the cell centre and the closest main road
Distance to the closest river	m	Distance between the cell centre and the closest river
Distance to urban areas	m	Distance between the cell centre and the closest urban area
Distance to protected areas	m	Distance between the cell centre and the closest protected area

**Table 3:** Estimates of relative contributions of the environmental variables. Permutation importance represents, for each environmental variable in turn, the resulting drop in training AUC when the values of that variable on training presence and background data are randomly permuted, normalised to show percentages. Values are averages of replicate runs.

Variable	Permutation Importance
Distance to woods	14.4
Urban areas	14.2
Open cultivated areas	11.9
Closed cultivated areas	10.5
Distance to rivers	8
Distance to protected areas	7.7
Distance to urban areas	7
Open natural areas	6.6
Shrublands	6.3
Wood cover	6.2
Human population density, distance to main road	<6

## Data Availability

The data are available upon reasonable request from the corresponding author.

## Supplementary Materials

The following supporting information can be downloaded at: https://www.mdpi.com/article/10.3390/ani15040546/s1. Table S1: Reclassified land cover classes.
