# Supplementary material for: Can the Wolf (Canis lupus) Thrive in Highly Anthropised Lowlands? First Habitat Suitability Analysis of the Po Plain, Italy
Source: Animals (Basel). 2025 Feb 13;15(4):546. doi: 10.3390/ani15040546 (PMC11851539; doi:10.3390/ani15040546)
Supplement: Supplementary file 1 [file animals-15-00546-s001.zip › animals-3444443-supplementary.pdf]

Supplementary Material

Can the Wolf Stably Occupy Highly Anthropized Lowlands? First Habitat Suitability Analysis of the Po Plain, Italy

Table S1. Reclassified land cover classes.

Reclassified Land Cover Classes	CLC Classes	CLC Code
Open cultivated areas	Non-irrigated arable land	2.1.1
	Permanently irrigated land	2.1.2
	Rice fields	2.1.3
	Vineyards	2.2.1
	Fruit trees and berry plantation	2.2.2
	Annual crops associated with permanent crops	2.4.1
	Complex cultivation patterns	2.4.2
Closed cultivated areas	Land principally occupied by agriculture with significant areas of natural vegetation	2.4.3
	Agro-forestry areas	2.4.4
Open natural areas	Pastures	2.3.1
	Natural grassland	3.2.1
	Moors and heathland	3.2.2
	Sparsely vegetated area	3.3.3
Urban areas	Continuous urban fabric	1.1.1
	Discontinuous urban fabric	1.1.2
	Industrial or commercial units	1.2.1
	Road and rail networks and associated land	1.2.2
	Port areas	1.2.3
	Airports	1.2.4
	Dump sites	1.3.2
	Construction sites	1.3.3
	Green urban areas	1.4.1
	Sport and leisure facilities	1.4.2
Forest cover	Broad-leaved forests	3.1.1
	Coniferous forests	3.1.2
	Mixed forests	3.1.3
Shrublands	Sclerophyllous vegetation	3.2.3
	Transitional woodland-shrubs	3.2.4
Other	Beaches, dunes, sands	3.3.1
	Inland marshes	4.1.1
	Peat bogs	4.1.2
	Water bodies	5.1.2
	Estuaries	5.2.2
